# OPA1-Exon4b Binds to mtDNA D-Loop for Transcriptional and Metabolic Modulation, Independent of Mitochondrial Fusion

**DOI:** 10.3389/fcell.2020.00180

**Published:** 2020-04-09

**Authors:** Liang Yang, Haite Tang, Xiaobing Lin, Yi Wu, Sheng Zeng, Yongzhang Pan, Yukun Li, Ge Xiang, Yi-Fang Lin, Shi-Mei Zhuang, Zhiyin Song, Yiguo Jiang, Xingguo Liu

**Affiliations:** ^1^CAS Key Laboratory of Regenerative Biology, Joint School of Life Sciences, Hefei Institute of Stem Cell and Regenerative Medicine, Guangzhou Institutes of Biomedicine and Health, Chinese Academy of Sciences, Guangzhou Medical University, Guangzhou, China; ^2^Guangzhou Regenerative Medicine and Health Guangdong Laboratory, Guangdong Provincial Key Laboratory of Stem Cell and Regenerative Medicine, South China Institute for Stem Cell Biology and Regenerative Medicine, Institute for Stem Cell and Regeneration, Guangzhou Institutes of Biomedicine and Health, University of Chinese Academy of Sciences, Chinese Academy of Sciences, Guangzhou, China; ^3^State Key Laboratory of Respiratory Disease, Guangzhou Regenerative Medicine and Health Guangdong Laboratory, Guangzhou Institutes of Biomedicine and Health, Chinese Academy of Sciences, Guangzhou, China; ^4^MOE Key Laboratory of Gene Function and Regulation, School of Life Sciences, Collaborative Innovation Center for Cancer Medicine, Sun Yat-sen University, Guangzhou, China; ^5^Hubei Key Laboratory of Cell Homeostasis, College of Life Sciences, Wuhan University, Wuhan, China; ^6^State Key Laboratory of Respiratory Disease, The First Affiliated Hospital of Guangzhou Medical University, Guangzhou, China

**Keywords:** Optic Atrophy 1 (OPA1), mitochondrial DNA, mitochondrial fusion, hepatocellular carcinoma, mtDNA D-loop

## Abstract

Optic Atrophy 1 (OPA1) has well-established roles in both mitochondrial fusion and apoptotic crista remodeling and is required for the maintenance and distribution of mitochondrial DNA (mtDNA), which are essential for energy metabolism. However, the relationship between OPA1 and mitochondrial metabolism and the underlying mechanisms remain unclear. Here, we show that OPA1-Exon4b modulates mitochondrial respiration and rescues inner mitochondrial membrane potential (Δψm), independent of mitochondrial fusion. OPA1-Exon4b is required for the maintenance of normal TFAM distribution and enhances mtDNA transcription by binding the D-loop of mtDNA. Finally, we show that mRNA levels of OPA1 isoforms containing Exon4b are specifically downregulated in hepatocellular carcinoma (HCC), leading to a reduction in Δψm. Thus, our study demonstrates a novel mitochondrial functional self-recovery pathway involving enhanced mtDNA transcription-mediated recovery of mitochondrial respiratory chain proteins. This mitochondrial fusion-independent pathway may contribute to mitochondrial multi-functional switches in tumorigenesis.

## Introduction

Mitochondria contain their own DNA, which is organized in discrete structures called nucleoids and spread within the mitochondrial network (Amati-Bonneau et al., 2008; Bogenhagen et al., 2008). Nucleoid proteins include not only factors involved in replication and transcription but also structural proteins required for the maintenance of mitochondrial DNA (mtDNA) (Brown et al., 2011). Nucleoids are reported to be tethered to the inner mitochondrial membrane (IMM) by a series of DNA–protein and protein–protein interactions (Chen et al., 2003; Chan, 2006). Super-resolution fluorescence microscopy techniques have been used to reveal the structure of nucleoids, which are closely associated with IMM and appear to be wrapped around the cristae or the crista-like inner membrane invaginations. Nucleoids differ greatly in size and shape, and exhibit in concave, split, or amorphous forms. Nucleoids co-localize with mitochondrial transcription factor A (TFAM) and mtDNA polymerase gamma (POLG) (Chen et al., 2007). Freely diffusible mitochondrial matrix proteins are found to be largely excluded from the nucleoid (Chen et al., 2010).

Maintenance and distribution of mtDNA are essential for mtDNA stability, energy metabolism, and mitochondrial lineage. Mounting evidence suggests that the mtDNA integrity can be affected by mitochondrial dynamics, including mitochondria fusion and fission. These mitochondrial dynamics also play a role in maintaining normal mitochondrial metabolic function, as well as the regulatory roles in cell signaling and differentiation (Coller et al., 2001; Delettre et al., 2001; Elachouri et al., 2011; Cui et al., 2013; Del Dotto et al., 2017; Farge and Falkenberg, 2019). Indeed, studies in neuronal (Frezza et al., 2006; Folmes et al., 2013) and muscular cells (Frilling et al., 2010) demonstrate that mitochondrial dynamics-related proteins, such as dynamin-related protein 1 (DRP1) and mitofusins (MFNs), contribute to the integrity and distribution of mtDNA. Optic Atrophy 1 (OPA1), a dynamin-related protein of IMM, functions in both IMM fusion and cristae maintenance (Gilkerson et al., 2008). *OPA1* mutations were reported to induce the accumulation of mtDNA deletions in skeletal muscle (Griparic et al., 2007; He et al., 2012). Furthermore, *OPA1* silencing led to mtDNA depletion, a phenomenon related to replication inhibition and distribution alteration of mtDNA (Chen et al., 2003). These findings lead to the hypothesis that OPA1 might contribute to the attachment of nucleoid to IMM.

OPA1 is encoded by a complicated set of at least eight mRNA variants that are specified by the presence of exons 4, 4b, or 5b (Holt et al., 2007; Hudson et al., 2008). OPA1 Exon4b is conserved throughout evolution and is involved in the maintenance of Δψm and mitochondrial fusion (Holt et al., 2007). OPA1 isoforms containing Exon4b such as OPA1 isoform 5 (OPA1-iso5) are cleaved into shorter isoforms by Yme1L, leading to an imbalance of long and short isoforms and thus to inhibition of mitochondrial fusion (Ishikawa et al., 2008; Kukat et al., 2015). Whether OPA1 is associated with mitochondrial metabolism and the underlying mechanisms are unclear.

Hepatocellular carcinoma (HCC) is one of the five most common cancers worldwide, and the 5-year survival rate of patients diagnosed with HCC is less than 10% (Lee et al., 2005). Western blotting evaluation of HCC samples and matched non-tumor tissue samples demonstrates that OPA1 expression is decreased in up to 40% of HCC patients (Legros et al., 2004), suggesting important roles for OPA1 in the development of HCC. In the present study, we demonstrate that Exon4b controls the transcription regulation of mtDNA and mitochondrial metabolic maintenance via maintaining TFAM distribution, a process conserved in HCC cells SK Hep1.

## Results

### OPA1-Exon4b Rescues Δψm Independent of Mitochondrial Fusion

**FIGURE 1:**
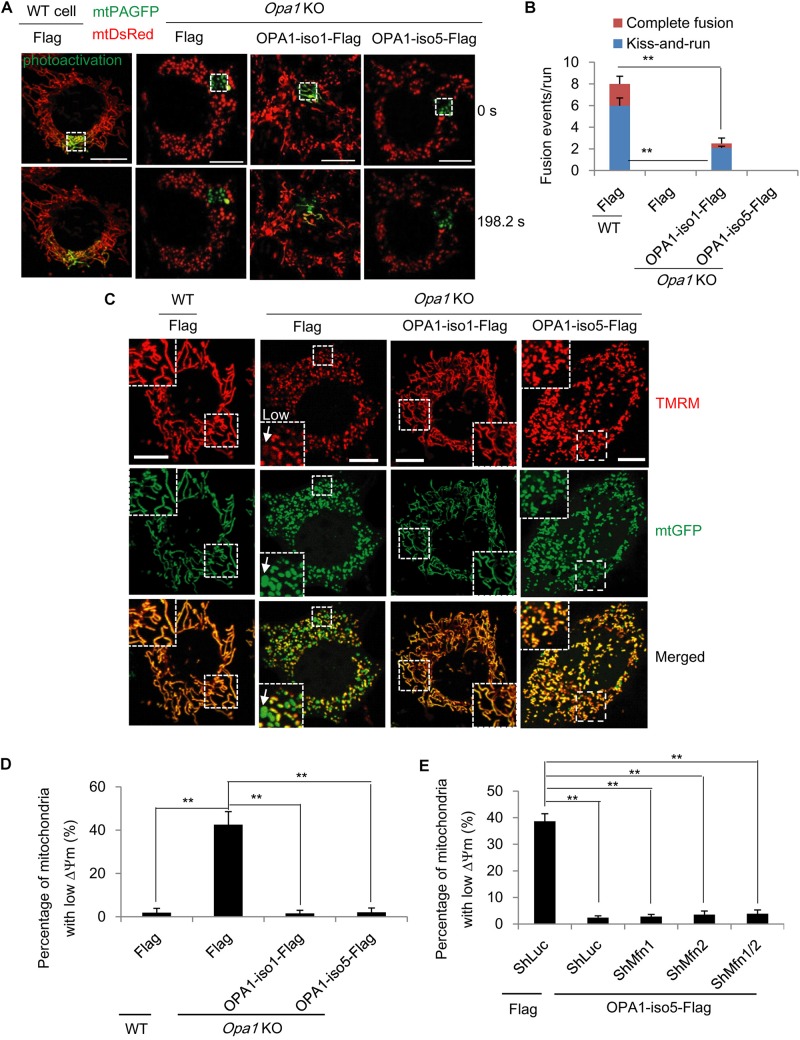
OPA1-Exon4b is not required for mitochondrial fusion but ensues mitochondrial bioenergetic recovery. **(A)** Labeling of one subset of mitochondria by photoactivation of PAGFP in cells expressing both mtPAGFP and mtDsRed, as indicated on the top (scale bar: 10 μm). **(B)** Quantitation of mitochondrial fusion events, including kiss-and-run and complete fusion, per run (*n* = 10 runs). **(C)** Δψm of WT MEF cells expressing Flag and *Opa1* KO cells expressing Flag, OPA1-iso1-Flag, or OPA1-iso5-Flag. The mean TMRM FI of mitochondria in WT cells was normalized to 1. The TMRM FI per mitochondrion below 0.3 denoted low (white arrow). **(D)** Quantitation of percentage of mitochondria with low Δψm in WT MEF cells expressing Flag and *Opa1* KO cells expressing Flag, OPA1-iso1-Flag, or OPA1-iso5-Flag (≥20 cells for three biological replicates). **(E)** Quantitation of percentage of mitochondria with low Δψm in *Opa1* KO cells expressing ShLuc plus Flag and *Opa1* KO cells expressing OPA1-iso5-Flag plus ShLuc, Sh*Mfn*1, Sh*Mfn*2 or Sh*Mfn*1/2 (≥20 cells for three biological replicates). **p < 0.01, one-way ANOVA.

It has been known that bioenergetics of dysfunctional mitochondria can be restored by heteroplasmic mitochondrial fusion, leading to exchange of mtDNA nucleoids (Liu and Hajnoczky, 2011) or respiratory chain proteins (Liu et al., 2009). Given the regulatory role of OPA1 on IMM fusion, we first assessed the effect of OPA1-Exon4b on mitochondrial fusion. As the OPA1-iso5 differs from OPA1 isoform 1 (OPA1-iso1) only by its presence of OPA1-Exon4b, we investigated the roles of OPA1-Exon4b on mitochondrial fusion by complementing *Opa1* knockout (KO) mouse embryonic fibroblast (MEF) cells with either OPA1-iso5 or OPA1-iso1. After the OPA1 expression levels in wild type (WT) MEF cells expressing Flag and *Opa1* KO cells expressing Flag, OPA1-iso1, or OPA1-iso5 were confirmed by western blotting (Supplementary Figure 1A), we co-expressed mitochondrial matrix-targeted photoactivatable green fluorescent protein (mtPAGFP) and mtDsRed in WT cells expressing Flag and *Opa1* KO cells expressing Flag, OPA1-iso1-Flag, or OPA1-iso5-Flag. As reported previously (Coller et al., 2001), high-resolution time-lapse confocal microscopy with region-of-interest scanning was employed to selectively and irreversibly photoactivate subpopulations of mitochondria (Figure 1A). In this assay, fusion events were classified as either complete or kiss-and-run by monitoring mitochondrial dynamics after photoactivation (Coller et al., 2001). As expected, *Opa1* KO cells expressing Flag didn’t show any fusion events. Expression of OPA1-iso1 restored both complete (*p* = 1.46398E-08) and kiss-and-run (*p* = 0.0002) fusion events. In stark contrast, expression of OPA1-iso5 failed to rescue the fusion defects of *Opa1* KO cells, indicating that the presence of Exon4b in OPA1-iso5 impedes mitochondrial fusion (Figures 1A,B and Supplementary Figures 1B,C).

Next, we measured Δψm in WT MEF cells expressing Flag and *Opa1* KO cells expressing Flag, OPA1-iso1-Flag, or OPA1-iso5-Flag by tetramethyl rhodamine methyl ester (TMRM, Invitrogen, United States) staining. We observed that, consistent with two previous reports (McBride et al., 2006; Liu et al., 2009), in *Opa1* KO cells but not WT cells, 42.5 ± 3.7% of mitochondria were depolarized, showing low Δψm (*p* = 0.0004), as judged by a TMRM FI ratio of less than 0.3, relative to normal mitochondria. As expected, expression of OPA1-iso1 reduced the percentage of mitochondrial with low Δψm (*p* = 0.0023), in agreement with its ability to rescue mitochondrial fusion (Figures 1C,D). OPA1-iso5 is constitutively cleaved into short isoforms by Yme1L and, as such, is not involved in IMM fusion (Figure 1B). Surprisingly, we observed that expression of OPA1-iso5 recovered Δψm to the same extent that OPA1-iso1 did (*p* = 0.0005) (Figures 1C,D). Then, we asked how the Exon4b-containing OPA1-iso5 could recover Δψm in the absence of IMM fusion. We first tested the possibility that OPA1-Exon4b recovered Δψm by mitochondrial outer membrane (OMM) fusion. We silenced the expression of *Mfn1* (*p* = 0.0067) and *Mfn2* (*p* = 0.0035) necessary for OMM fusion in *Opa*1 KO cells expressing OPA1-iso5-Flag (Supplementary Figures 1D,E). We observed that Δψm was maintained in *Opa1* KO MEF cells expressing OPA1-iso5 even after silencing *Mfn1*, *Mfn2*, or *Mfn1/2* (Figure 1E and Supplementary Figure 1F). Therefore, OPA1-Exon4b recovered Δψm not mainly dependent on OMM fusion.

### OPA1-Exon4b Partly Rescues Mitochondrial Respiration

**FIGURE 2:**
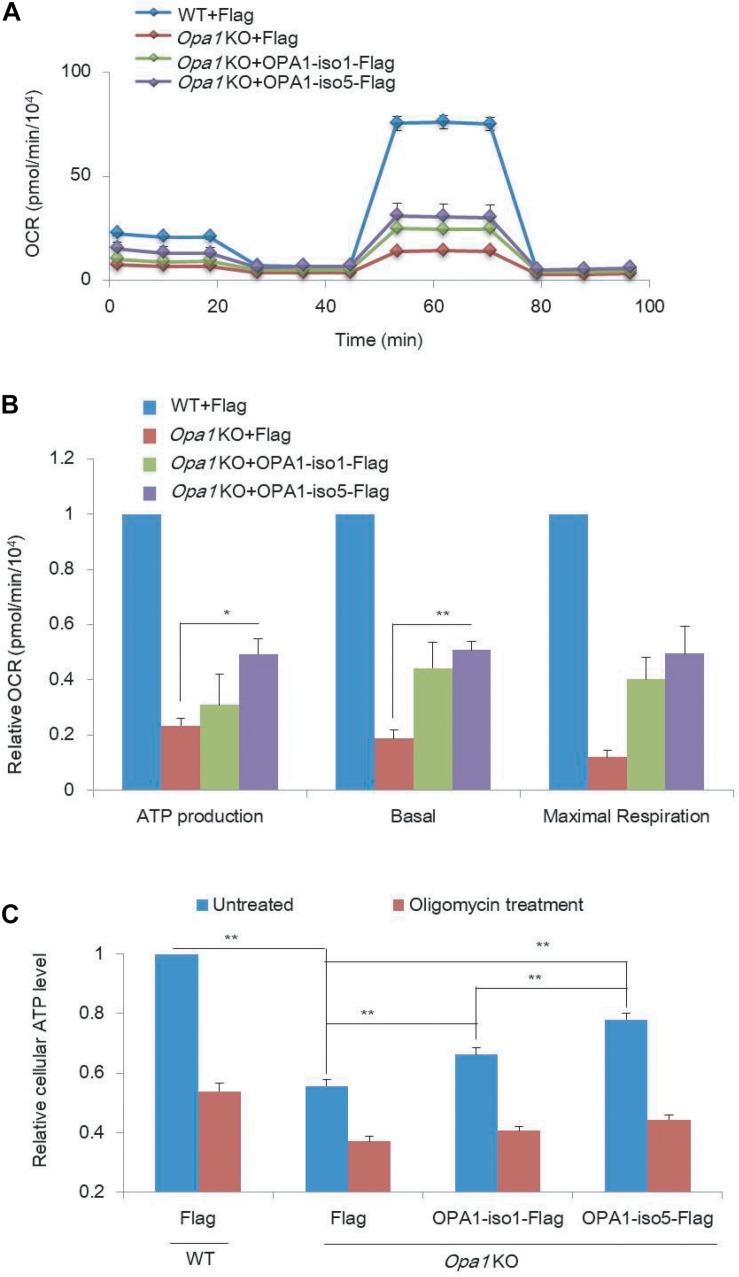
OPA1 Exon-4b and Exon4 partially recover mitochondrial respiration activity. **(A)** OCR measurements in WT MEF cells expressing Flag and *Opa1* KO cells expressing Flag, OPA1-iso1-Flag, or OPA1-iso5-Flag. **(B)** Relative ATP production, basal, and maximal respiration using OCR measurements in WT MEF cells expressing Flag and *Opa1* KO cells expressing Flag, OPA1-iso1-Flag or OPA1-iso5-Flag (*n* = 2, biological replicates). WT MEF cells were used as control, whose ATP production, basal, and maximal respiration were normalized to 1. **(C)** Cellular ATP levels in WT MEF cells expressing Flag and *Opa1* KO cells expressing Flag, OPA1-iso1-Flag, or OPA1-iso5-Flag, with or without oligomycin treatment (*n* = 3, biological replicates). *p < 0.05, **p < 0.01, one-way ANOVA.

Besides Δψm, we asked how mitochondrial respiration activity is affected by OPA1-Exon4b. We measured oxygen consumption rate (OCR) and extracellular acidification rate (ECAR) in WT MEF cells expressing Flag and *Opa1* KO cells expressing Flag, OPA1-iso1-Flag, or OPA1-iso5-Flag. *Opa1* KO cells exhibited significantly lower basal OCR (*p* = 0.0176), with an increase in ECAR, compared to that of WT MEF cells. *Opa1* KO cells expressing either OPA1-iso1 or OPA1-iso5 displayed higher levels of basal OCR (*p* = 0.0124) and ATP production (*p* = 0.0422) than *Opa1* KO cells (Figures 2A,B and Supplementary Figures 2A–C), suggesting improved mitochondrial function. We further measured cellular and mitochondrial ATP production and found that *Opa1* KO cells expressing either OPA1-iso1 (*p* = 0.0039 for cellular ATP and *p* = 0.0501 for mitochondrial ATP) or OPA1-iso5 (*p* = 0.0003 for cellular ATP and *p* = 0.0059 for mitochondrial ATP) displayed higher levels of mitochondrial and cellular ATP than *Opa1* KO cells. *Opa1* KO cells expressing OPA1-iso5 displayed higher cellular (*p* = 0.0033) and mitochondrial ATP (*p* = 0.0402) levels and a lower ECAR than those expressing OPA1-iso1 (Figure 2C). Considering the possible impact of cell apoptosis and viability on ATP production, we measured cell apoptosis by flow cytometry and detected cell viability using CCK8 assay in WT MEF cells expressing Flag and *Opa1* KO cells expressing Flag, OPA1-iso1-Flag, or OPA1-iso5-Flag. The results showed that cell apoptosis and viability were not altered by OPA1 knockout or rescue (Supplementary Figures 2D,E). All these results indicate that OPA1-Exon4b partly rescues mitochondrial respiration. OPA1-iso5 overexpression also enhanced cellular ATP level in Hela (*p* = 0.0147) and 293T (*p* = 0.0090) cells (Supplementary Figures 2G,H).

### OPA1-Exon4b Maintains Normal TFAM Distribution

**FIGURE 3:**
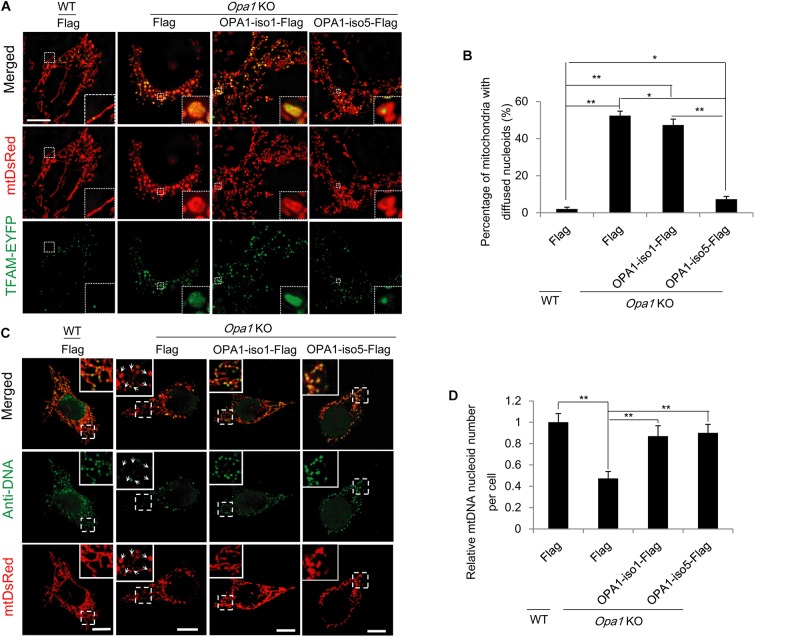
OPA1-Exon4b maintains normal TFAM distribution. **(A)** N-SIM Images of TFAM distribution in WT MEF cells expressing Flag and *Opa1* KO cells expressing Flag, OPA1-iso1-Flag, or OPA1-iso5-Flag. **(B)** Quantification of the percentage of mitochondria with diffuse TFAM in panel **(A)** (≥15 cells for three biological replicates, scale bar: 10 μm). **(C)** Anti-DNA immunofluorescence in WT MEF cells expressing Flag and *Opa1* KO cells expressing Flag, OPA1-iso1-Flag, or OPA1-iso5-Flag (scale bar: 10 μm). **(D)** Quantification of mtDNA nucleoid number per cell in WT MEF cells expressing Flag and *Opa1* KO cells expressing Flag, OPA1-iso1-Flag, or OPA1-iso5-Flag (≥20 cells for three biological replicates). *p < 0.05, **p < 0.01, one-way ANOVA.

TFAM packs mtDNA into mitochondrial nucleoids that are required for mtDNA transcription and replication (Olichon et al., 2007; Parone et al., 2008), which is necessary for the maintenance of normal mitochondrial function (e.g., respiration and ATP production). Therefore, we investigated the effect of OPA1-Exon4b on TFAM distribution and the mtDNA nucleoid number. We applied TFAM-EYFP in combination with mtDsRed to visualize TFAM distribution by a Nikon structured illumination microscopy (N-SIM). While most mitochondria (98.0 ± 1.0%) in WT MEF cells showed normal punctate structures of TFAM, a large proportion of mitochondria (52.3 ± 2.5%) in *Opa1* KO cells showed diffuse TFAM. Notably, in cells expressing OPA1-iso5 but not OPA1-iso1, the proportion of mitochondria with diffuse TFAM was decreased to 7.3 ± 0.2% (*p* = 0.0106) (Figures 3A,B and Supplementary Figure 3A). We also detected the number of mtDNA nucleoids by Anti-DNA IF and mtDNA copy number by qPCR and found that *Opa1* KO cells expressing either OPA1-iso5 (*p* = 1.85742E-06 for mtDNA nucleoid number and *p* = 0.0034 for mtDNA copy number) or OPA1-iso1 (*p* = 0.0106 for mtDNA nucleoid number and *p* = 0.0141 for mtDNA copy number) had more mtDNA nucleoids and mtDNA copy number than *Opa1* KO cells expressing Flag (Figures 3C,D and Supplementary Figure 3B), while overexpression of OPA1-iso5 or OPA1-iso1 didn’t increase mtDNA nucleoid number in Hela and 293T cells (Supplementary Figures 3C,D). These results indicate that, though both OPA1 iso1 and iso5 could rescue mtDNA nucleoid number and mtDNA copy number, OPA1 iso5, but not iso1, maintains TFAM puncture structure, suggesting that Exon4b contributes largely to the normal TFAM distribution by its interaction with TFAM (Chen et al., 2003).

### OPA1-Exon4b Binds mtDNA D-Loop Region and Increases mtDNA Transcription

**FIGURE 4:**
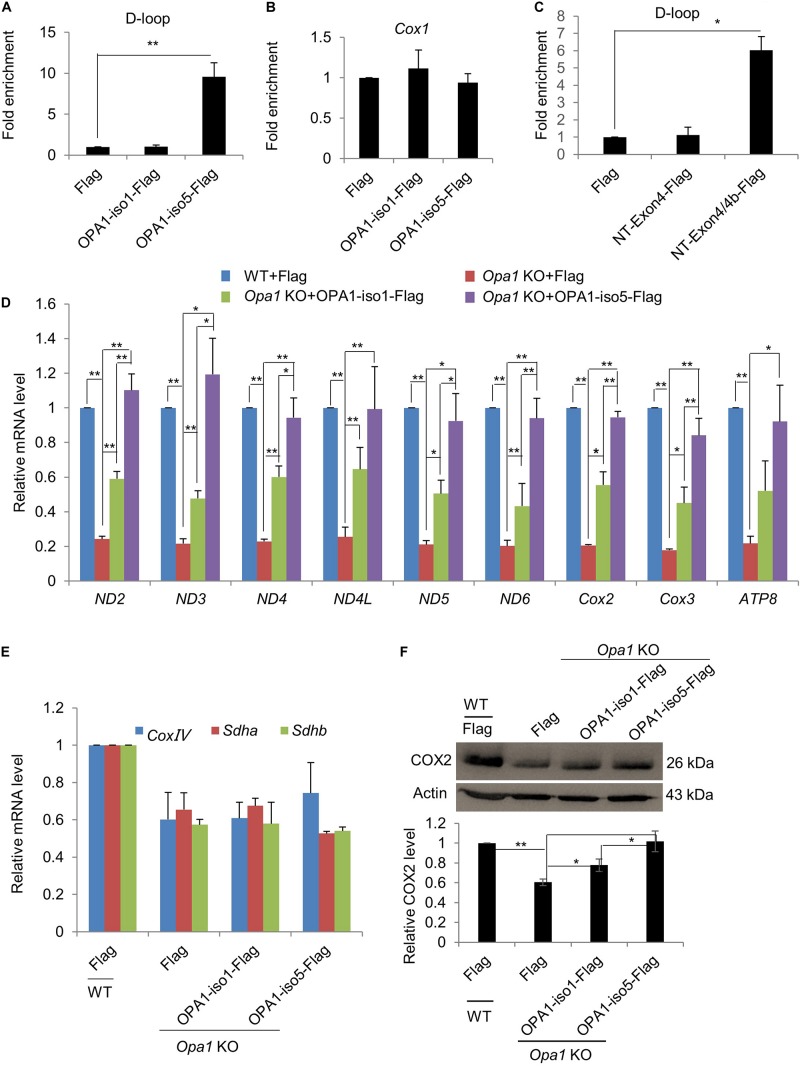
OPA1-Exon4b modulates mtDNA transcription. **(A,B)** Anti-Flag ChIP was carried out using WT MEF cells expressing Flag and *Opa1* KO cells expressing Flag, OPA1-iso1-Flag, or OPA1-iso5-Flag. The precipitated DNA was analyzed by qPCR using primer pairs for the D-loop region **(A)** or *Cox1* **(B)**. *n* = 3, biological replicates. **(C)** Anti-Flag ChIP was carried out using WT MEF cells expressing Flag and *Opa1* KO cells expressing Flag, NT-Exon4-Flag, or NT-Exon4/4b-Flag. The precipitated DNA was analyzed by qPCR using primer pairs for the D-loop region. *n* = 3, biological replicates. **(D)** Relative mRNA levels of 9 mtDNA genes in WT MEF cells expressing Flag and *Opa1* KO cells expressing Flag, OPA1-iso1-Flag, or OPA1-iso5-Flag (*n* = 3, biological replicates). **(E)** Relative mRNA levels of three nuclear genes encoding respiratory subunits in WT MEF cells expressing Flag and *Opa1* KO cells expressing Flag, OPA1-iso1-Flag or OPA1-iso5-Flag (*n* = 3, biological replicates). **(F)** Western blotting analysis of mtDNA-encoded Cox2 in WT MEF cells expressing Flag and *Opa1* KO cells expressing Flag, OPA1-iso1-Flag or OPA1-iso5-Flag. Band densities were quantified using ImageJ, and relative band densities are shown on the bottom. *n* = 3, biological replicates. *p < 0.05, **p < 0.01, one-way ANOVA.

The observation that OPA1-Exon4b maintained normal TFAM distribution (Figures 2A,B) led us to investigate its role in mtDNA transcription. Given the importance of D-loop in mtDNA transcription (Qiao et al., 2017), we first investigated whether OPA1-Exon4b could interact with the D-loop region of mtDNA. To this end, we performed anti-FLAG chromatin immunoprecipitation (ChIP) in *Opa1* KO cells expressing Flag, OPA1-iso1-Flag, or OPA1-iso5-Flag, and then detected the copy number of D-loop by quantitative PCR (qPCR). The results showed that OPA1-iso5, but not OPA1-iso1, interacted with the D-loop (*p* = 0.0083) (Figure 4A). To verify the specificity of the D-Loop binding of OPA1-Exon4b, we checked the binding of OPA1-iso5 with another region of mtDNA, i.e., the *Cox1* region. Importantly, OPA1-iso5 did not bind to the *Cox1* region (Figure 4B), supporting the specific binding of OPA1-Exon4b to the D-loop. N-terminal (NT)-Exon4/4b, a small hydrophobic 10-kDa peptide, generated by cleavage of OPA1-iso5 (Chen et al., 2003), was reported to interact with mtDNA nucleoids. We assessed whether NT-OPA1-Exon4/4b could interact with the D-loop using NT-OPA1-Exon4 as a control. We performed anti-Flag ChIP-qPCR in *Opa1* KO cells expressing NT-OPA1-Exon4-Flag, or NT-OPA1-Exon4/4b-Flag, and observed that NT-OPA1-Exon4/4b-Flag showed more interaction with the D-loop region than NT-OPA1-Exon4-Flag (*p* = 0.0118) (Figure 4C). Therefore, OPA1-Exon4b can bind to mtDNA D-loop region in a specific fashion.

The binding of OPA1-Exon4b to mtDNA D-loop led us to further assess the effect of Exon4b on mtDNA transcriptional regulation. We detected by qPCR transcriptional levels of nine mtDNA genes in WT MEF cells expressing Flag and *Opa1* KO cells expressing Flag, OPA1-iso1-Flag, or OPA1-iso5-Flag. All the tested genes showed significantly decreased expression in *Opa1* KO cells compared to the WT cells. Expression of both OPA1-iso1 and OPA1-iso5 restored the transcription of the tested genes in *Opa1* KO cells. Notably, OPA1-iso5 delivered a stronger effect than OPA1-iso1 (Figure 4D), indicating an important role for Exon4b in the regulation of mtDNA transcription. OPA1-iso5 also increased mtDNA transcription in Hela and 293T cells (Supplementary Figures 4A,B). We further analyzed transcription levels of three nuclear DNA-encoded respiratory chain subunits, such as *Sdha.* The expression of all the tested genes was reduced in *Opa1* KO cells, compared to that of WT cells. More importantly, expression of OPA1-iso1 or OPA1-iso5 failed to restore transcription of these nuclear genes in *Opa1* KO cells (Figure 4E). Finally, we assessed whether the effect on mtDNA transcription could alter the levels of proteins, we detected COX2 and SDHA by western blotting (Figure 4F and Supplementary Figure 4C). In agreement with the qPCR analysis, expression of both OPA1-iso5 (*p* = 0.0140) and OPA1-iso1 (*p* = 0.0253) increased the level of COX2, but not that of SDHA. Moreover, the effect of OPA1-iso5 was stronger than OPA1-iso1 (*p* = 0.0358). Taken together, OPA1-Exon4b binds the D-loop region and increases mtDNA transcription.

### Downregulation of Exon4b-Containing OPA1 Isoforms in HCC

**FIGURE 5:**
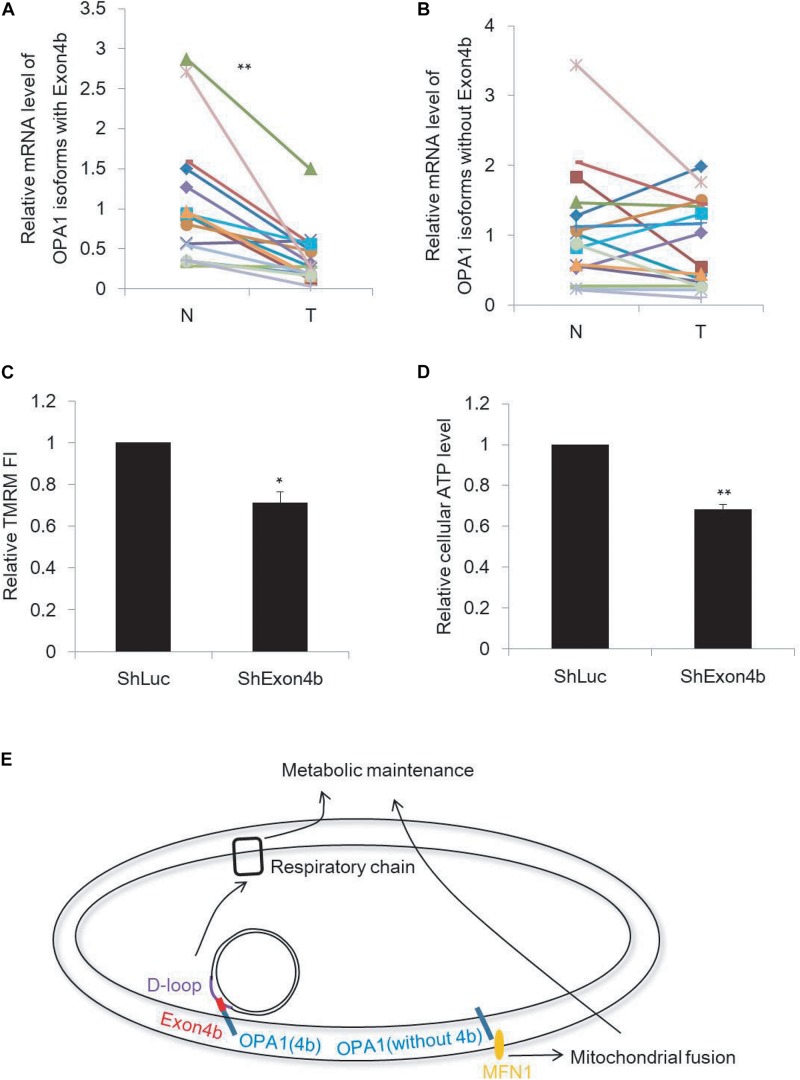
Downregulation of Exon4b-containing OPA1 isoforms in HCC. **(A,B)** mRNA levels of OPA1 isoforms with Exon4b **(A)** and OPA1 isoforms without Exon4b **(B)** in 22 paired HCC (T) and adjacent non-tumor (N) liver tissues (*n* = 22, paired-samples *t*-test **p < 0.01). **(C,D)** Relative TMRM fluorescence intensity (FI) (**C,** *n* = 3, biological replicates) and cellular ATP level (**D,** *n* = 3, biological replicates) in SK-Hep1 cells expressing ShExon4b compared to that in SK-Hep1 cells expressing ShLuc which is normalized to 1 (independent-samples *t*-test. *p < 0.05, **p < 0.01). **(E)** Model of the functions of OPA1 isoforms with or without Exon4b.

In cancer cells, energy is generated mainly through aerobic glycolysis, but not through mitochondrial respiration. Considering the essential roles of Exon4b on mitochondrial function including energetics, we tested whether the compromised mitochondrial respiration observed in some cancer cells could be associated with OPA1-Exon4b. We measured by qPCR the mRNA levels of Exon4b-containing OPA1 isoforms (i.e., isoforms 3, 5, 6, and 8) and those without Exon4b (i.e., isoforms 1, 2, 4, and 7) in 22 paired HCC and adjacent non-tumor liver tissues. We observed that both tissues expressed comparable levels of OPA1 isoforms without Exon4b (*p* = 0.4403). In contrast, the Exon4b-containing OPA1 isoforms were markedly decreased in tumor tissues (*p* = 0.0007, Figures 5A,B). This pointed to the correlation between the downregulation of OPA1-Exon4b and HCC tumorigenesis. Given this finding, we further tested the function of OPA1 Exon4b in human HCC cell line (SK-Hep1) by short hairpin RNA (shRNA)-mediated Exon4b silencing. After Exon4b silencing was validated by qPCR (*p* = 0.0009) and Western blotting (*p* = 0.0322) (Supplementary Figures 5A,B), we measured Δψm by TMRM staining and cellular ATP level by luciferase assay. We observed that silencing of Exon4b resulted in a significant decrease in Δψm (*p* = 0.0107) and cellular ATP (*p* = 0.0018) (Figures 5C–D). Hence, downregulation of OPA1 Exon4b is associated with compromised mitochondrial function in HCC.

## Discussion

We showed here that OPA1-Exon4b is required for the maintenance of normal TFAM distribution and enhances mtDNA transcription by binding the D-loop of mtDNA. Two non-coding regions (NCRs), i.e., the major and minor NCR, are present in mtDNA. The major NCR, also known as the D-loop, is a region of 900-bp fragment containing transcription promoters of the heavy/light strands and the origin of replication of the heavy strand. Thus, the D-loop is essential for mtDNA replication and transcription. Interestingly, Exon4b was found to interact with the D-loop region of mtDNA specifically, providing an alternative explanation for the regulatory role of Exon4b-containing OPA1-iso5 on mtDNA transcription.

Surprisingly, OPA1 Exon4b, without fusion activity, recovers mitochondrial bioenergetics in Opa1 KO cells. Thus, we demonstrated how OPA1 isoforms bidirectionally control mitochondrial metabolic recovery via fusion dependent and independent manner. OPA1 isoforms containing Exon4b were shown to be fully cleaved by i-AAA protease Yme1L into short forms (Kukat et al., 2015). A recent study showed that OPA1 short forms were shown to restore energetic efficiency (Reyes et al., 2011). Here, we found that OPA1 Exon4b could not only maintain TFAM distribution but also bind with mtDNA D-loop, conceivably leading to an enhanced expression of mtDNA-encoded respiratory proteins and thus the recovery of bioenergetics. Based on these, we propose a novel model of mitochondrial recovery involving the enhancement of mtDNA transcription (Figure 5E).

We also assessed the role of OPA1-Exon4b in mitochondrial bioenergetics of cancer cells. We found that the level of OPA1-Exon4b was downregulated in HCC tumor tissues and that Exon4b silencing compromised Δψm in an HCC cell line. These results suggest that the loss of function of Exon4b may be linked to the HCC tumorigenesis. Studies have demonstrated important roles for mitochondrial metabolism in tumorigenesis. For instance, the well-known Warburg effect describes that cancer cells derive their energy from glucose fermentation yielding lactate even in the presence of oxygen, despite the fact that they have higher energy needs. The Warburg effect is also characterized by the malfunction of mitochondria. Previous studies have shown that mtDNA point mutation and its content reduction may play a role in the regulation of mitochondrial function in various cancer cells including HCC (Song et al., 2007; Scarpulla, 2008; Suen et al., 2008; Tatsuta and Langer, 2008; Tondera et al., 2009). Based on our results, we identify in this study that the decreased expression of Exon4b, necessary for optimal mitochondrial function (Figure 5E), may also account for the malfunction of mitochondria and thus establishment of the Warburg effect in cancer cells.

In summary, we have revealed that Exon4b is essential for the maintenance of TFAM distribution and contributes to mtDNA transcription through its binding with the D-loop region. In addition to the previous reported mitochondrial fusion-dependent pathway, we uncover here a novel fusion-independent mitochondrial function recovery pathway that is dependent on Exon4b (Figure 5E). The malfunction of this pathway may be linked to the establishment of the Warburg effect, which could play a role in tumorigenesis.

## Materials and Methods

### Cells

*Opa1* KO and control WT MEF cells were purchased from ATCC (Manassas, United States). Human HCC cells SK-Hep1 were obtained from Professor S-MZ (Sun Yat-sen University, China). Hela, 293T and Platinum-E cells were grown in Dulbecco’s modified Eagle’s medium (DMEM), supplemented with 10% fetal bovine serum (FBS), streptomycin (50 lg/ml), and penicillin (50 U/ml). All cultures were maintained at 37°C in a humidified incubator containing 5% CO_2_. For imaging experiments, cells were plated on glass coverslips.

### cDNA Samples of Human Tumor Tissue Specimens and Adjacent Non-tumor Tissues

cDNA samples of paired HCC and adjacent non-tumor liver tissues from patients undergoing HCC resection were obtained from the Cancer Center of Sun Yat-sen University in Guangzhou, China. None of the patients had received any local or systemic anticancer treatments before the surgery. Both tumor and non-tumor tissues were histologically confirmed. The protocol was approved by the Institute Research Ethics Committee at the Sun Yat−sen University Cancer Center (approval number: GZR2019-086) and informed consent was obtained from each patient. The patients were anonymously coded in accordance with local ethical guidelines, as instructed by the Declaration of Helsinki.

### Plasmid Constructs

All mitochondrial matrix-targeting fluorescent protein (mtFP) vectors encoded the targeting sequence of cytochrome c oxidase subunit VIII to achieve mitochondrial matrix localization. TFAM-EYFP plasmid was constructed by replacing the sequence encoding the cytochrome c oxidase subunit VIII of mtEYFP with that of mTfam (NM_009360). mtDsRed, mtPAGFP, and TFAM-EYFP were sub-cloned into the retroviral vector pMXs-Flag. The pMSCV-puro vectors expressing eight isoforms of human OPA1 were gifts from Professor ZS (Wuhan University, China). All these OPA1 isoforms were then sub-cloned into the retroviral vector pMXs-Flag. NT-Exon4-Flag and NT-Exon4/4b-Flag were cloned with the primers by adding a C-terminus Flag sequence as described (Chen et al., 2003). The reported target sequence for Exon4b and Exon4 ShRNA as described (Chen et al., 2003) were used as shRNA and constructed into the pSUPER vector (oligoengine, VEC-PRT-0002), and then, cells infected with pSUPER were selected with puromycin (Genomeditech, GM-040401-2; 2 μg/mL) for 48 h prior to sampling.

### Retrovirus Packaging

For virus production, 8 × 10^6^ Platinum-E cells were plated in a 10-cm dish for 24 h, and then transfected with 10 μg pMXs-based plasmid/40 μg Polyethylenimine (PEI, Polyscience Co., United States) in 1 mL Opti-MEM (Invitrogen, United States). The culture medium was replaced 12 h after transfection, and the medium containing retrovirus was collected 36 h later. Retrovirus generated using pMXs-Flag vector were used as a control to equalize the total amount of retrovirus administered to cells.

### Western Blotting

Equal amounts of total protein (∼20 μg) were resolved by 10% polyacrylamide/sodium dodecyl sulfate gel electrophoresis and then transferred onto polyvinylidene fluoride membranes. Membranes were then blocked for 1 h, followed by incubation with anti-SDHA (Abcam, 1:1,000), anti-Cox2 (Abcam, 1:1,000), anti-OPA1 (Abcam, 1:1,000), or anti-Actin (Santa Cruz, 1:2,000) antibodies. After incubation with the primary antibody, membranes were incubated with horseradish peroxidase-coupled secondary antibody and immunoreactivity was subsequently detected using Immobilon Western Chemiluminescent HRP Substrate (Millipore, United States).

### Live Cell Oxygen Consumption

OCR and ECAR were measured with the XF24 extracellular flux analyzer (Seahorse Biosciences) as described (Wang et al., 2013). WT cells expressing Flag and *Opa*1 KO cells expressing Flag, OPA1-iso1-Flag, or OPA1-iso5-Flag were seeded at a density of 50,000 cells per well of a XF24 cell culture microplate and incubated overnight to ensure attachment. Before measurement, cells were equilibrated for 1 h in XF base assay medium supplemented with 25 mM glucose, 1 mM sodium pyruvate, and 2 mM L-glutamine in a non-CO_2_ incubator. During the incubation time, we loaded 75 μL of 80 mM glucose, 9 μM oligomycin, and 1 M 2-deoxyglucose (for ECAR measurement) or 8 μM oligomycin, 9 μM FCCP, 10 μM rotenone, and 10 μM antimycin A (for OCR measurement), in XF assay media into the injection ports in the XF24 sensor cartridge. Each plotted value was normalized to total cells by counting cell number after measurements.

### ATP Measurement

Cellular ATP levels were determined using the ENLITEN ATP Assay System (Promega Corp., Madison, WI, United States). Cell extraction was performed with 2.5% trichloroacetic acid, and the sample was neutralized and diluted in 10 mM Tris-acetate (pH 7.75). ATP levels were then measured using the Luciferase/Luciferin reagent according to the manufacturer’s protocol. Mitochondrial ATP production was measured using a previous reported protocol (Yang L. et al., 2015) with a slight modification. Cells were treated with 10 μM oligomycin for 15 min before ATP levels were measured.

### Live Cell Microscopic Imaging

Imaging was performed with a Leica DMIRE2 inverted microscope (Leica Microsystems, Montreal, Germany) using a 100 × oil lens (Uapo340, NA 1.40) recording 1,024 × 1,024-pixel image. The Ar/ArKr laser was used for photoactivation of PAGFP at 458-nm and imaging of GFP at 488-nm excitation. The HeNe laser source was used for imaging of DsRed or TMRM at 543-nm excitation. PAGFP was photoactivated using the region-of-interest (ROI) scanning option in the Leica LAS AF Lite software. One 25 μm^2^ area was chosen per cell. 80 consecutive images were achieved every 5.83 s after photoactivation. Fusion events were classified as either complete fusion or kiss-and-run as described (Coller et al., 2001).

Δψm was measured by TMRM staining. For TMRM staining, cells were treated with 25 nM TMRM for 30 min and then replaced with 5 nM TMRM for imaging using the same confocal parameters.

### Apoptosis Assay

Cell apoptosis was analyzed using FITC Annexin V Apoptosis Detection Kit (BD Biosciences, 556547) according to the manufacturer’s protocol. Cells were washed twice with cold PBS and then resuspended in 1 × binding buffer. Cells were incubated with FITC Annexin V and PI for 15 min at room temperature in the dark. Then, cell suspension was treated with 1 × binding buffer and analyzed by flow cytometry using a BD Accuri C6 Plus flow cytometer (BD Biosciences) within 1 h, and data were analyzed by using FlowJo V10 software.

### Cellular Viability Assay

Cell viability was detected and quantified using a CCK8 assay kit (Beyotime, China). For CCK8 assay, cells were seeded into 96-well culture plate at a density of 2 × 10^3^ cells/well. After 6 h, 10 μL of CCK8 solution was added to each well and incubated for 1 h at 37°C. Then, the viability was recorded based on the optical density (OD) value detected at 450 nm.

### mtDNA Nucleoids Imaging by SIM

Cells overexpressing mtDsRed and TFAM-EYFP were seeded on coverslips and cultured for 24 h. Then, cells were fixed and mounted in slides and imaged by N-SIM (Nikon, Japan). The images were taken with a dual-color (laser 488 nm and laser 561 nm) SIM mode, using a 100 × oil (NA 1.49) objective with autofocus maintained by the Nikon Perfect Focus system. All images were reconstructed to maximum projections using NIS-Elements AR software (Nikon, Japan).

### Immunofluorescence

Cells were fixed with 4% paraformaldehyde for 15 min, washed, and permeabilized in 0.5% Triton X-100 for 15 min. Cells were then washed, blocked with 1% bovine serum albumen for 15 min, and incubated with primary antibody for 1 h. After washing, cells were then incubated with corresponding secondary antibody (Pierce, United States) for 1 h. All washes were with PBS and all procedures were performed at room temperature. Primary antibodies used were anti-DNA (Millipore, United States, 1:100).

### ChIP-qPCR

ChIP-qPCR was done following a previously reported protocol (Yang R.F. et al., 2015) with some modifications. Cells were cross-linked with 1% formaldehyde for 10 min at room temperature, then washed once with ice-cold PBS, and then harvested by scraping with a spatula. Cells were lysed in SDS buffer [1% SDS, 50 mM Tris–HCl (pH 8.0), 10 mM EDTA, and protease inhibitor cocktail] for 10 min at 4°C and sheared into 200–500-bp DNA fragments by sonication. ChIP-grade anti-Flag and control mouse IgG were purchased from Santa Cruz. The primers (forward, 5′-TCAAATGCGTTATCGCCC-3′; reverse, 5′-TTTCATGCCTTGACGGCT-3′) were specific for the D-loop region of mouse mtDNA (GenBank: *AB033825.1*).

### qPCR Analysis

Total RNA was extracted using Trizol reagent (Invitrogen, United States) according to the manufacturer’s instructions, and cDNA was synthesized by reverse transcription of 1 μg total RNA per sample using the ReverTra Ace^@^ kit (Toyobo, Japan). qPCR was performed using a CFX-96 real-time PCR detection system (Bio-Rad, United States) in conjunction with SsoAdvanced Universal SYBR Green Supermix (Bio-Rad, United States) using the following conditions: an initial denaturation step of 95°C for 30 s, followed by 40 cycles of denaturation at 95°C for 5 s, and annealing-elongation at 60°C for 20 s. The primers for detecting *Opa1* mRNA and different variant abundances in human samples were used as described (Holt et al., 2007). The primers for detecting mice and human mtDNA genes were used as described (Zhao et al., 2013). Amplification of β-actin cDNA in the same samples was used as an internal control for all PCR amplification reactions, and the primers (forward, 5-TGACGTGGACATCCGCAAAG-3; reverse, 5-CTGGAAGGTGGACAGCGAGG-3) were used to detect β-actin. Gene expression values were calculated based on the comparative quantitative method (the DDCT method) and normalized to values obtained from the amplification of β-actin. For mtDNA copy number determination, total DNA was extracted by a TIANamp Genomic DNA Kit (Tiangen, DP304–03), and the primers for detecting ND5 were used to detect mtDNA copy number.

### Statistics

The data were shown as mean ± standard deviation (SD), and all experiments were repeated at least three times. All statistical tests were two-sided and performed using SPSS software (SPSS/IBM Inc., Chicago, IL, United States). All data meet normal distribution and have uniform standard deviations. Paired-sample *t*-test (Figures 5A,B) and independent-sample *t*-test (Figure 5C) were used for comparisons between two groups. For three or more groups, one-way ANOVA (Figures 1–4) was used firstly to detect the difference among these groups; if the *P*-value was less than 0.05, and then multiple comparisons were performed using least significant difference (LSD) *t*-test to detect the difference between any two groups. *P*-value less than 0.05 is considered as significant, while value less than 0.01 is considered as highly significant.

## Data Availability Statement

All datasets generated for this study are included in the article/Supplementary Material.

## Ethics Statement

The studies involving human participants were reviewed and approved by the Institute Research Ethics Committee at the Sun Yat−sen University Cancer Center (approval number: GZR2019-086). The patients/participants provided their written informed consent to participate in this study.

## Author Contributions

All authors listed have made a substantial, direct and intellectual contribution to the work, and approved it for publication.

## Conflict of Interest

The authors declare that the research was conducted in the absence of any commercial or financial relationships that could be construed as a potential conflict of interest.

## Supplementary Material

FIGURE S1 OMM fusion is not required for Δψm recovery mediated by OPA1-Exon4b, related to Figure 1. **(A)** Western blotting analysis of OPA1 and Actin of WT MEF cells expressing Flag and Opa1 KO cells expressing Flag, OPA1-iso1-Flag, or OPA1-iso5-Flag. Band densities were quantified using ImageJ, and relative band densities are shown on the right. *n* = 3, biological replicates. **(B,C)** Time course of a typical mitochondrial kiss-and-run event **(B)** or complete fusion event **(C)**, as determined by photoactivation assay (Scale bar: 1 μm). **(D)** Detection of protein expression levels of *Mfn1* in WT MEF cells expressing ShMfn1 with ShLuc as control. *n* = 3, biological replicates. Band densities were quantified using ImageJ, and relative band densities are shown on the bottom. *n* = 3, biological replicates. **(E)** Detection of protein expression levels of *Mfn2* in WT MEF cells expressing ShMfn2 with ShLuc as control. *n* = 3, biological replicates. Band densities were quantified using ImageJ, and relative band densities are shown on the bottom. *n* = 3, biological replicates. (F) Measurement of Δψm of mitochondria with low Δψm in *Opa1* KO cells expressing OPA1-iso5-Flag plus ShLuc, ShMfn1, ShMfn2, or ShMfn1/2 and OPA1 KO cells expressing ShLuc plus Flag (≥10 cells for each group with three biological replicates). ^∗^*p* < 0.05; ^∗∗^*p* < 0.01, one-way ANOVA.

FIGURE S2 OPA1 Exon-4b and Exon4 modulate mitochondrial respiration, related to Figure 2. **(A,B)** Basal and spare respiratory capacity **(A)**, proton leak, and ATP production **(B)** in WT MEF cells expressing Flag and *Opa1* KO cells expressing Flag, OPA1-iso1-Flag, or OPA1-iso5-Flag obtained from Figure 2A. **(C–E)** Glycolysis, glycolytic capacity, and glycolytic reserve calculated from ECAR measurements **(C)**, apoptotic cells by flow cytometry **(D)**, and cell viability by CCK-8 (**E**, *n* = 3, biological replicates) in WT MEF cells expressing Flag and *Opa1* KO cells expressing Flag, OPA1-iso1-Flag, or OPA1-iso5-Flag. OPA1 protein expression by western blotting **(F)** and relative cellular ATP level (**G**, *n* = 3, biological replicates) in Hela and 293T cells that expressed Flag, OPA1-iso1-Flag, or OPA1-iso5-Flag. ^∗^*p* < 0.05, ^∗∗^*p* < 0.01, one-way ANOVA.

FIGURE S3 mtDNA nucleoid imaging in *Opa1* KO cells, Hela and 293T cells that overexpressed OPA1-iso1 or OPA1-iso5, related to Figure 3. **(A)** *N*-SIM images of diffuse TFAM in 3 *Opa1* KO cells expressing OPA1-iso1-Flag from Figure 3A. **(B)** Relative mtDNA copy number in WT MEF cells expressing Flag and Opa1 KO cells expressing Flag, OPA1-iso1-Flag, or OPA1-iso5-Flag (*n* = 3, biological replicates). **(C)** Anti-Tfam IF in Hela and 293T cells that overexpressed OPA1-iso1 or OPA1-iso5. **(D)** Quantification mtDNA nucleoid number of B (≥15 cells for three biological replicates).

FIGURE S4 OPA1-iso5 increased mtDNA transcription, related to Figure 4. **(A,B)** Relative mRNA levels of mtDNA genes in Hela and 293T cells expressing Flag, OPA1-iso1-Flag, or OPA1-iso5-Flag (*n* = 3, biological replicates). **(C)** Western blotting analysis of nuclear-encoded SDHA in WT MEF cells expressing Flag and *Opa1* KO cells expressing Flag, OPA1-iso1-Flag or OPA1-iso5-Flag. Band densities were quantified using ImageJ, and relative band densities are shown on the bottom. *n* = 3, biological replicates. ^∗^*p* < 0.05, ^∗∗^*p* < 0.01, one-way ANOVA.

FIGURE S5 Exon4b silencing in SK-Hep1 cells, related to Figure 5. Detection of mRNA levels of OPA1 isoforms with Exon4b **(A)** and protein level of OPA1 **(B)** in SK-Hep1 cells expressing ShExon4b with ShLuc as control. Band densities of B were quantified using ImageJ, and relative band densities are shown on the bottom. *n* = 3, biological replicates. ^∗∗^*p* < 0.01, one-way ANOVA.
