# Supplementary figures and images for: OPA1-Exon4b Binds to mtDNA D-Loop for Transcriptional and Metabolic Modulation, Independent of Mitochondrial Fusion
Source: Front Cell Dev Biol. 2020 Apr 9;8:180. doi: 10.3389/fcell.2020.00180 (PMC7179665; doi:10.3389/fcell.2020.00180)

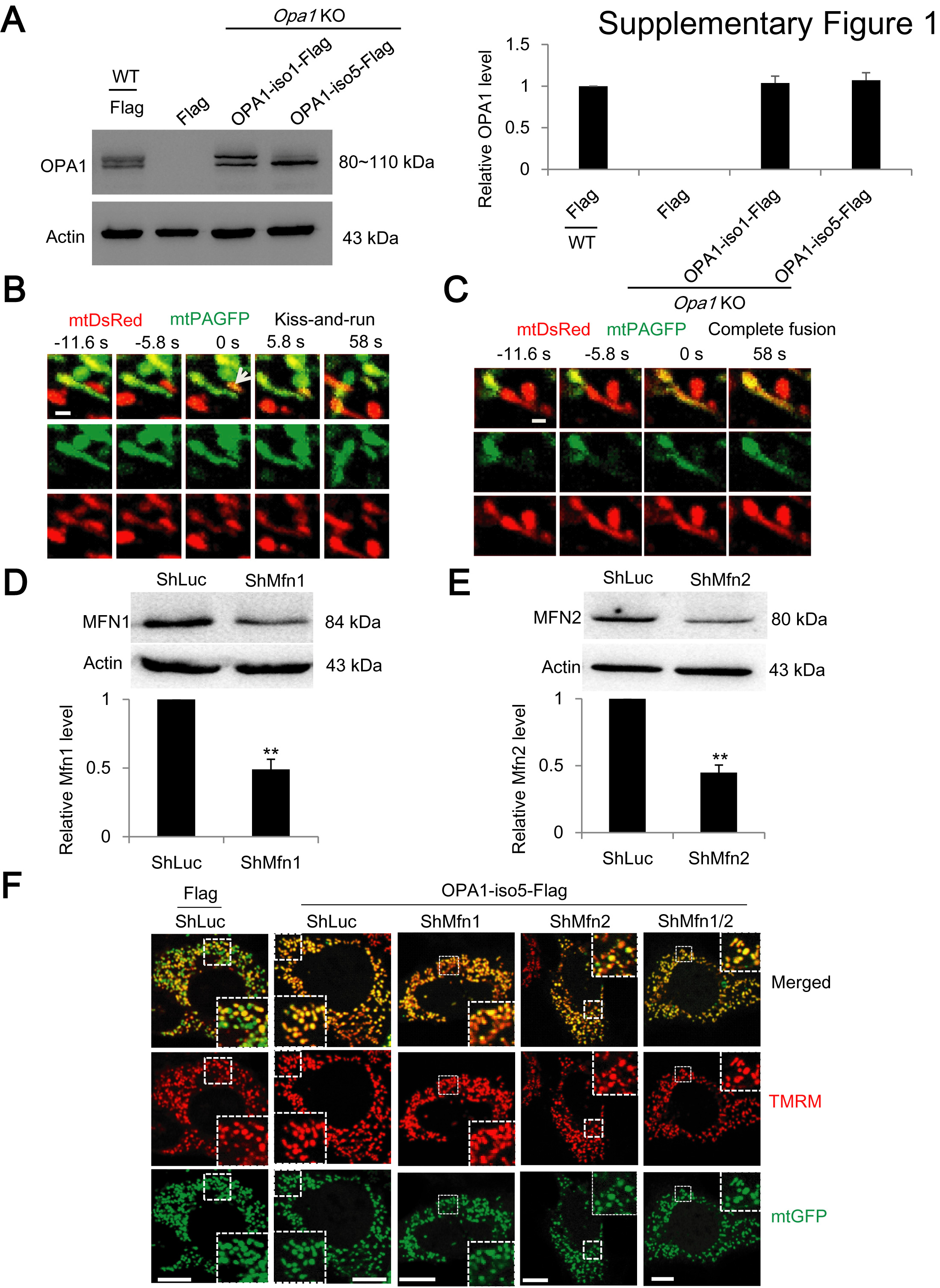

Supplement: FIGURE S1 — OMM fusion is not required for Δψm recovery mediated by OPA1-Exon4b, related to Figure 1. (A) Western blotting analysis of OPA1 and Actin of WT MEF cells expressing Flag and Opa1 KO cells expressing Flag, OPA1-iso1-Flag, or OPA1-iso5-Flag. Band densities were quantified using ImageJ, and relative band densities are shown on the right. n = 3, biological replicates. (B,C) Time course of a typical mitochondrial kiss-and-run event (B) or complete fusion event (C), as determined by photoactivation assay (Scale bar: 1 μm). (D) Detection of protein expression levels of Mfn1 in WT MEF cells expressing ShMfn1 with ShLuc as control. n = 3, biological replicates. Band densities were quantified using ImageJ, and relative band densities are shown on the bottom. n = 3, biological replicates. (E) Detection of protein expression levels of Mfn2 in WT MEF cells expressing ShMfn2 with ShLuc as control. n = 3, biological replicates. Band densities were quantified using ImageJ, and relative band densities are shown on the bottom. n = 3, biological replicates. (F) Measurement of Δψm of mitochondria with low Δψm in Opa1 KO cells expressing OPA1-iso5-Flag plus ShLuc, ShMfn1, ShMfn2, or ShMfn1/2 and OPA1 KO cells expressing ShLuc plus Flag (≥10 cells for each group with three biological replicates). ∗p < 0.05; ∗∗p < 0.01, one-way ANOVA. [file Image_1.JPEG]

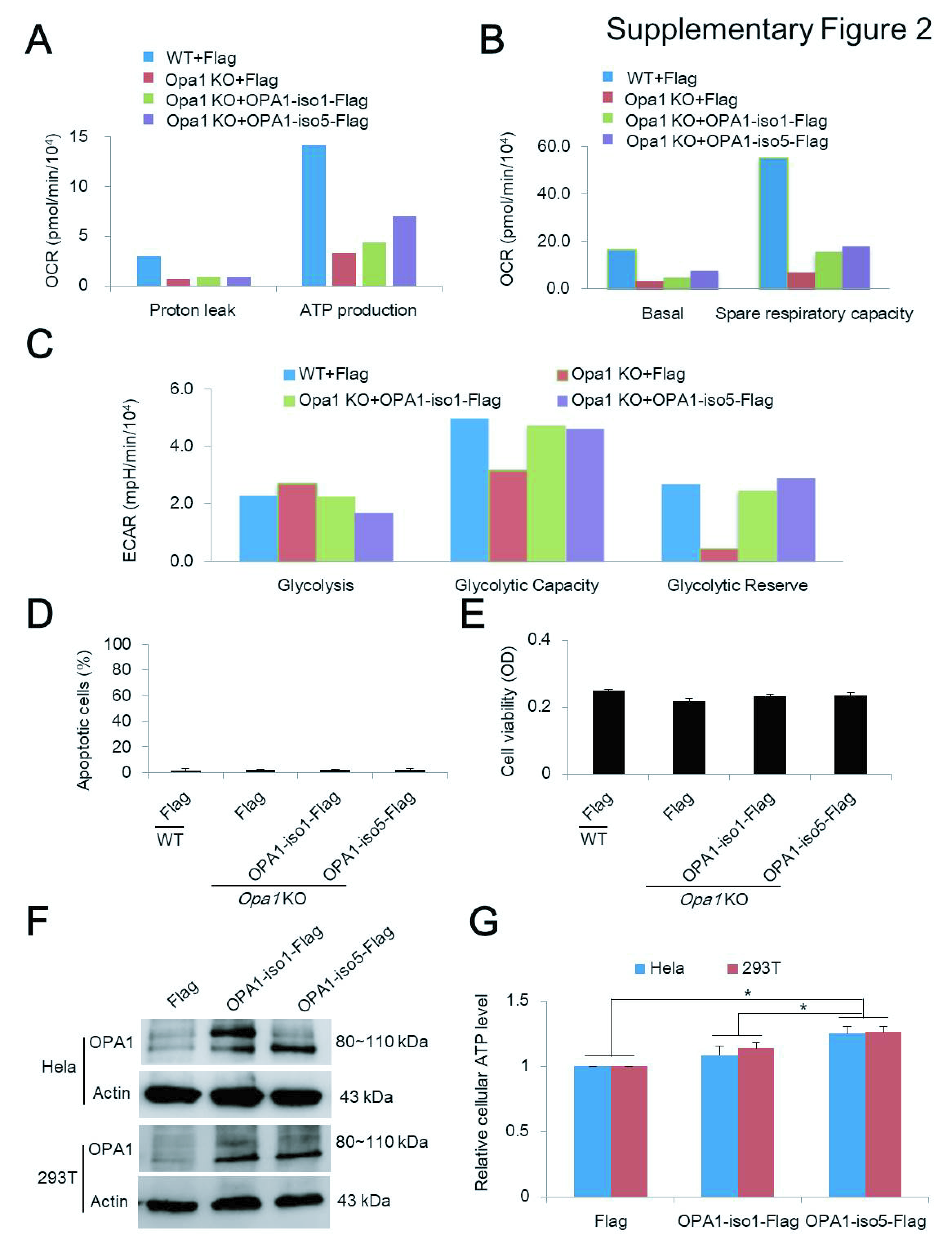

Supplement: FIGURE S2 — OPA1 Exon-4b and Exon4 modulate mitochondrial respiration, related to Figure 2. (A,B) Basal and spare respiratory capacity (A), proton leak, and ATP production (B) in WT MEF cells expressing Flag and Opa1 KO cells expressing Flag, OPA1-iso1-Flag, or OPA1-iso5-Flag obtained from Figure 2A. (C–E) Glycolysis, glycolytic capacity, and glycolytic reserve calculated from ECAR measurements (C), apoptotic cells by flow cytometry (D), and cell viability by CCK-8 (E, n = 3, biological replicates) in WT MEF cells expressing Flag and Opa1 KO cells expressing Flag, OPA1-iso1-Flag, or OPA1-iso5-Flag. OPA1 protein expression by western blotting (F) and relative cellular ATP level (G, n = 3, biological replicates) in Hela and 293T cells that expressed Flag, OPA1-iso1-Flag, or OPA1-iso5-Flag. ∗p < 0.05, ∗∗p < 0.01, one-way ANOVA. [file Image_2.JPEG]

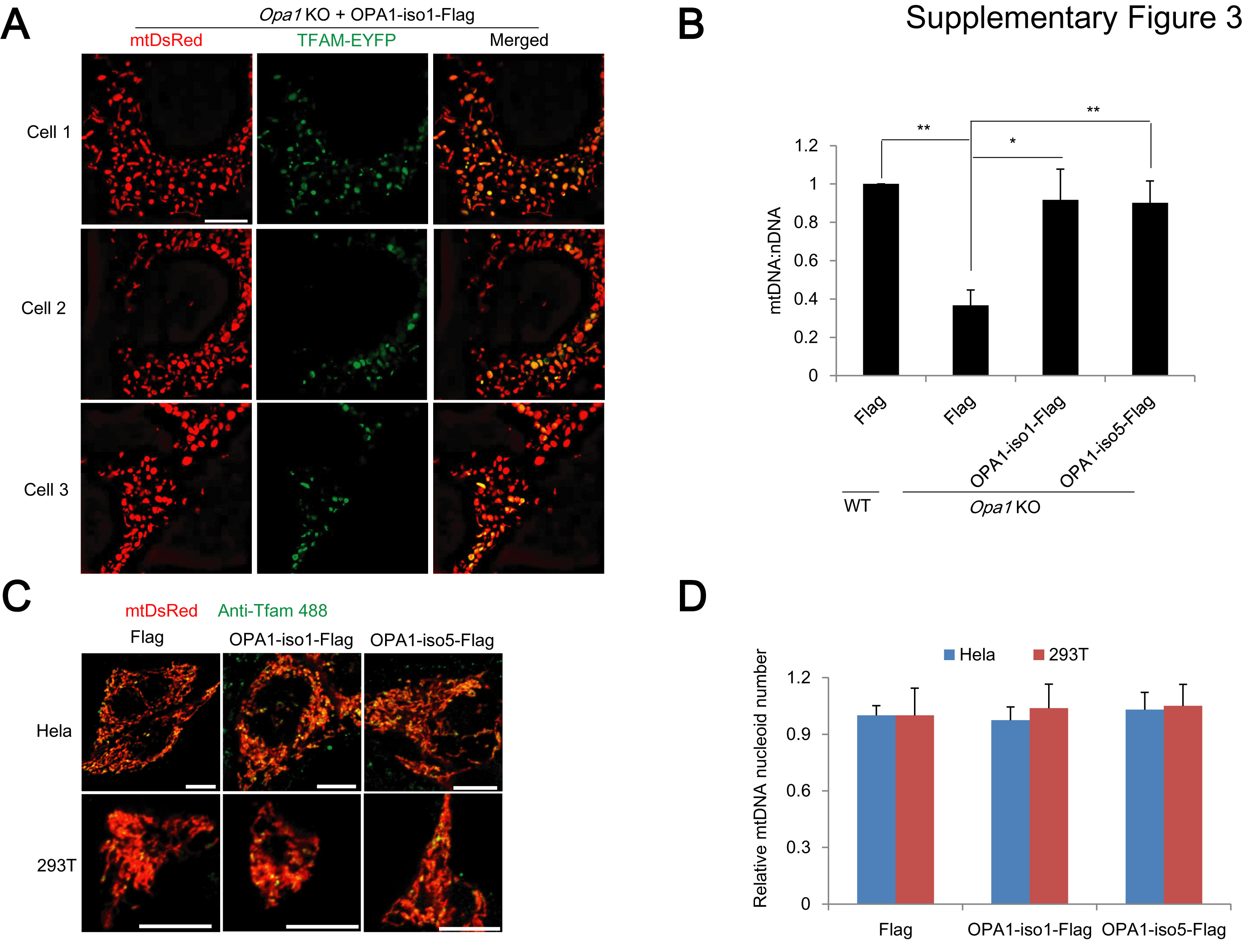

Supplement: FIGURE S3 — mtDNA nucleoid imaging in Opa1 KO cells, Hela and 293T cells that overexpressed OPA1-iso1 or OPA1-iso5, related to Figure 3. (A) N-SIM images of diffuse TFAM in 3 Opa1 KO cells expressing OPA1-iso1-Flag from Figure 3A. (B) Relative mtDNA copy number in WT MEF cells expressing Flag and Opa1 KO cells expressing Flag, OPA1-iso1-Flag, or OPA1-iso5-Flag (n = 3, biological replicates). (C) Anti-Tfam IF in Hela and 293T cells that overexpressed OPA1-iso1 or OPA1-iso5. (D) Quantification mtDNA nucleoid number of B (≥15 cells for three biological replicates). [file Image_3.JPEG]

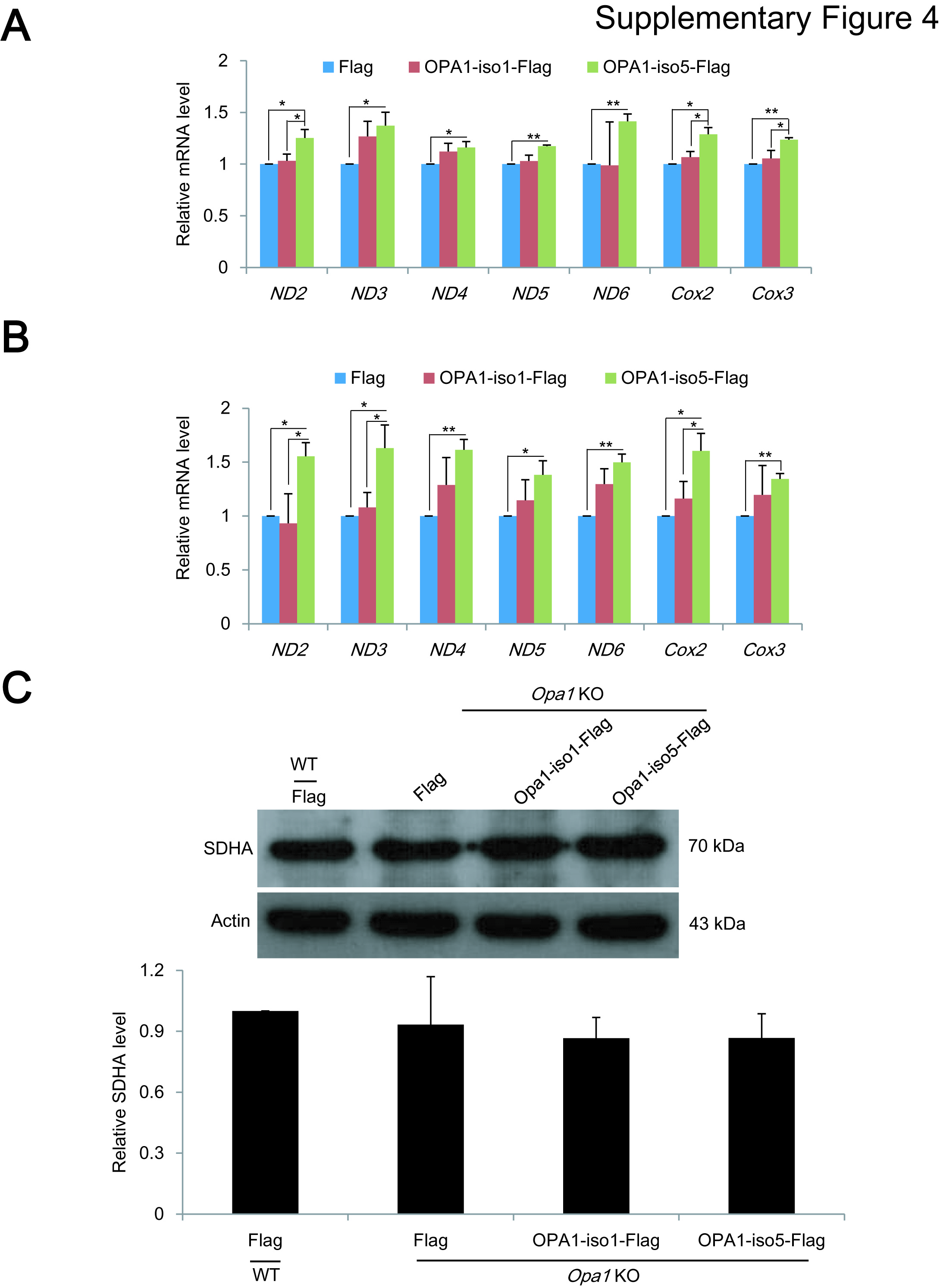

Supplement: FIGURE S4 — OPA1-iso5 increased mtDNA transcription, related to Figure 4. (A,B) Relative mRNA levels of mtDNA genes in Hela and 293T cells expressing Flag, OPA1-iso1-Flag, or OPA1-iso5-Flag (n = 3, biological replicates). (C) Western blotting analysis of nuclear-encoded SDHA in WT MEF cells expressing Flag and Opa1 KO cells expressing Flag, OPA1-iso1-Flag or OPA1-iso5-Flag. Band densities were quantified using ImageJ, and relative band densities are shown on the bottom. n = 3, biological replicates. ∗p < 0.05, ∗∗p < 0.01, one-way ANOVA. [file Image_4.JPEG]

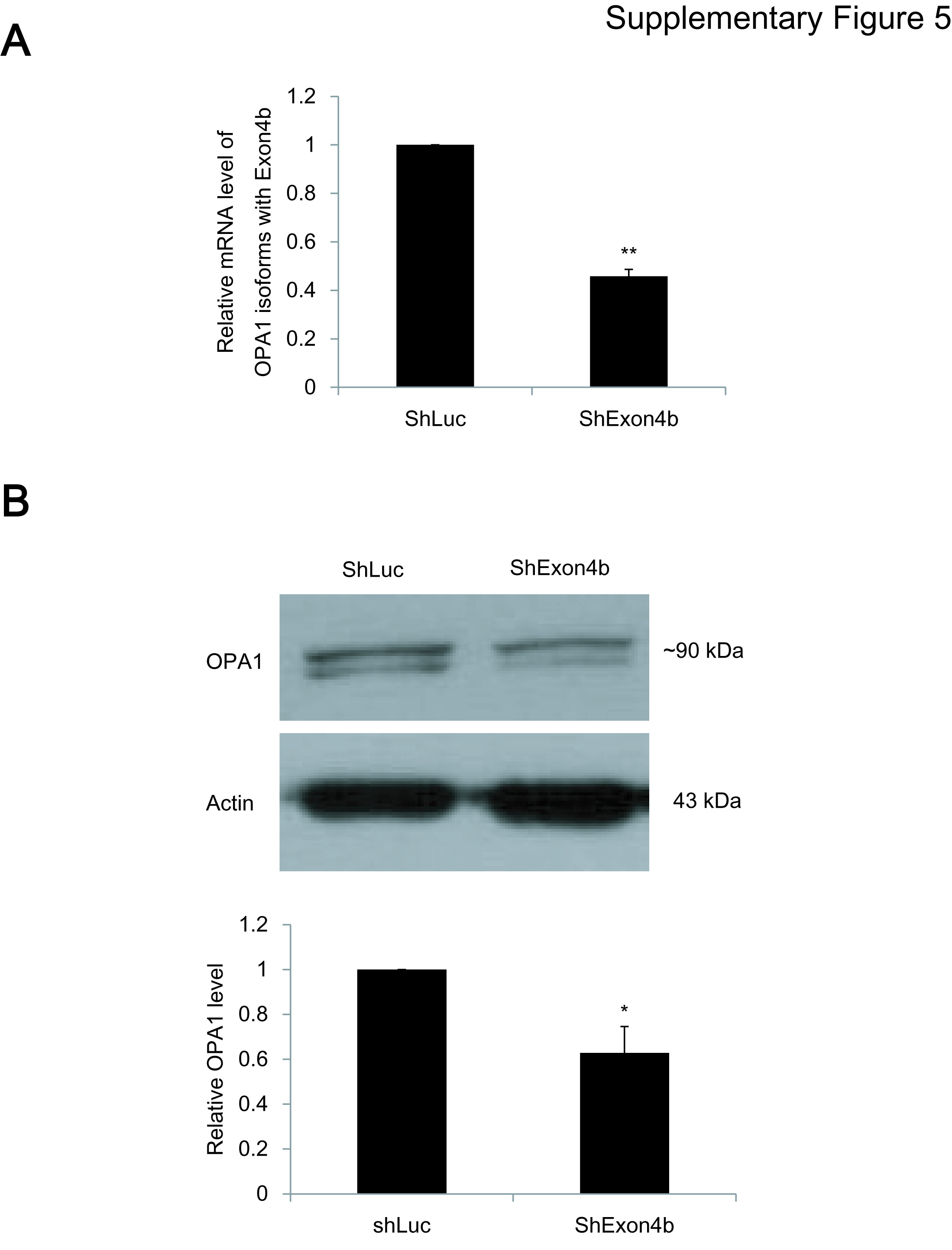

Supplement: FIGURE S5 — Exon4b silencing in SK-Hep1 cells, related to Figure 5. Detection of mRNA levels of OPA1 isoforms with Exon4b (A) and protein level of OPA1 (B) in SK-Hep1 cells expressing ShExon4b with ShLuc as control. Band densities of B were quantified using ImageJ, and relative band densities are shown on the bottom. n = 3, biological replicates. ∗∗p < 0.01, one-way ANOVA. [file Image_5.JPEG]
